# Supplementary material for: Conformational plasticity and allosteric communication networks explain Shelterin protein TPP1 binding to human telomerase
Source: Commun Chem. 2023 Nov 7;6:242. doi: 10.1038/s42004-023-01040-y (PMC10630336; doi:10.1038/s42004-023-01040-y)
Supplement: Supplementary file 2 — Supplementary Information [file 42004_2023_1040_MOESM2_ESM.pdf]

Supplementary Information

Simone Aureli[1,3,4], Vince Bart Cardenas[1], Stefano Raniolo[1] and Vittorio Limongelli[1,2,*]

[1] Faculty of Biomedical Sciences, Euler Institute, Università della Svizzera italiana, via G. Buffi 13, CH-6900 Lugano, Switzerland
[2] Department of Pharmacy, University of Naples “Federico II”, via D. Montesano 49, I-80131 Naples, Italy

[3] Current address: Institute of Pharmaceutical Sciences of Western Switzerland, University of Geneve, Rue Michel-Servet 1, CH-1211 Genève, Switzerland
[4] Current address: Swiss Institute of Bioinformatics, University of Geneva, CH-1206, Geneva, Switzerland.

* To whom correspondence should be addressed. Tel: +41586664293; Email: vittoriolimongelli@gmail.com

SUPPLEMENTARY MATERIAL

The data hereby reported are divided in the following sections:

- **Supplementary Note 1:** Details on WT TPP1, Glu169∆ TPP1, Lys170∆ TPP1, and Leu95Gln TPP1 MD simulations
- **Supplementary Note 2:** Intra-protein H-bonds in the Leu95Gln TPP1 MD simulation
- **Supplementary Note 3:** Complementary data on the time-series analysis
- **Supplementary Note 4:** Protein structure network of the WT TPP1, Glu169∆ TPP1, Lys170∆ TPP1, and Leu95Gln TPP1 monomers
- **Supplementary Note 5:** List of TPP1 mutations
- **Supplementary Note 6:** Additional data on the heterodimers MD simulations
- **Supplementary Note 7:** Contacts ruling the TPP1-TERT protein-protein interaction
- **Supplementary Note 8:** Details on systems building

Supplementary Note 1: Details on WT TPP1, Glu169Δ TPP1, Lys170Δ TPP1, Leu95Gln TPP1 MD simulations

In this section, we described supplementary information about the 12 MD simulations on the systems WT TPP1, Glu169Δ TPP1, Lys170Δ TPP1, Leu95Gln TPP1. In Tab. S1, we reported the pooled percentage of cluster families represented in Fig. 2. In Fig. S1, we displayed our analysis of the time evolution of the intra-protein contacts ruling the conformational plasticity of the TEL-patch, i.e. Glu171-Arg180, Glu169-Arg180, Glu168-Arg180, and Asp166-Ser210. As shown in Fig. S1, the MD simulations of Glu169Δ TPP1 and Lys170Δ TPP1 are marked by the establishment of lost-lasting interactions along the whole trajectories.

	WT TPP1	Glu169Δ TPP1	Lys170Δ TPP1	Leu95Gln TPP1
Cluster 1	19.00%	46.00%	44.67%	58.33%
Cluster 2	17.33%	16.67%	21.00%	10.67%
Cluster 3	10.00%	//	//	//

Tab. S1: Tables reporting the relevance of each cluster family in Fig. 2. The percentages have been calculated by merging the three replica of each system into a single trajectory. Additional details on the clusterization procedure are shown in the "Methods" section.

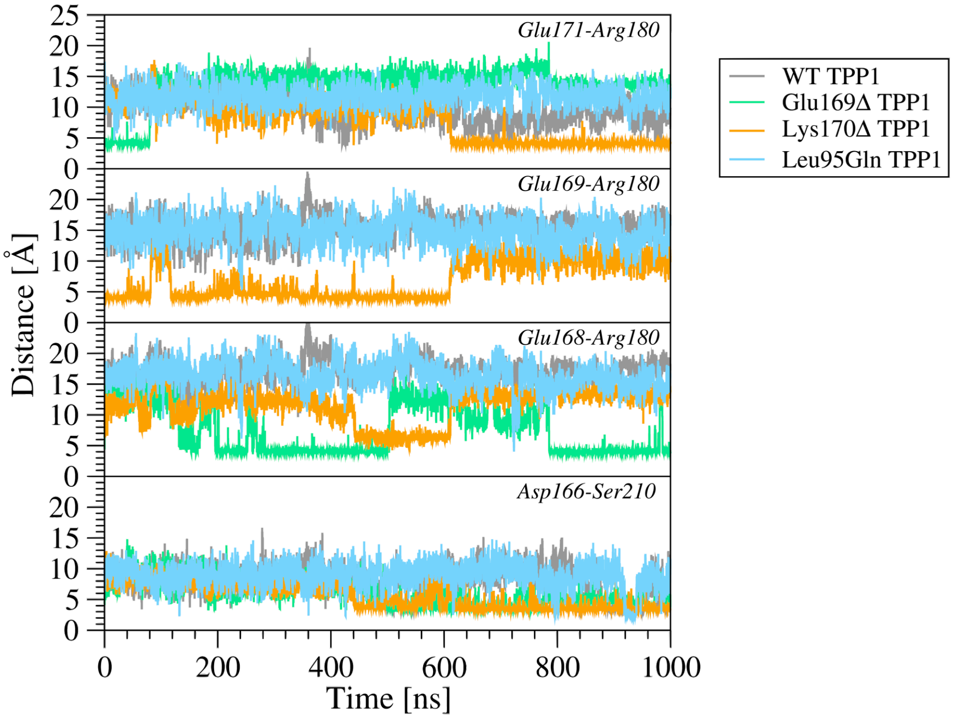

Fig. S1: Plot reporting the intra-protein distances ruling the conformational plasticity of the TEL-patch, i,e, Glu171-Arg180, Glu169-Arg180, Glu168-Arg180, and Asp166-Ser210. The interactions occurring during the WT TPP1 simulation are coloured in grey, the ones established in Glu169Δ TPP1 are coloured in orange, the ones established in Lys170Δ TPP1 are coloured in green, and the ones the ones established in Leu95Gln TPP1 are coloured in cyan.

Supplementary Note 2: Intra-protein H-bonds in the Leu95Gln TPP1 MD simulation

Herein, we reported additional details about the Leu95Gln TPP1 simulation. In particular, we show in Fig. S2 the intra-protein H-bonds that Gln95 establishes during the trajectory.

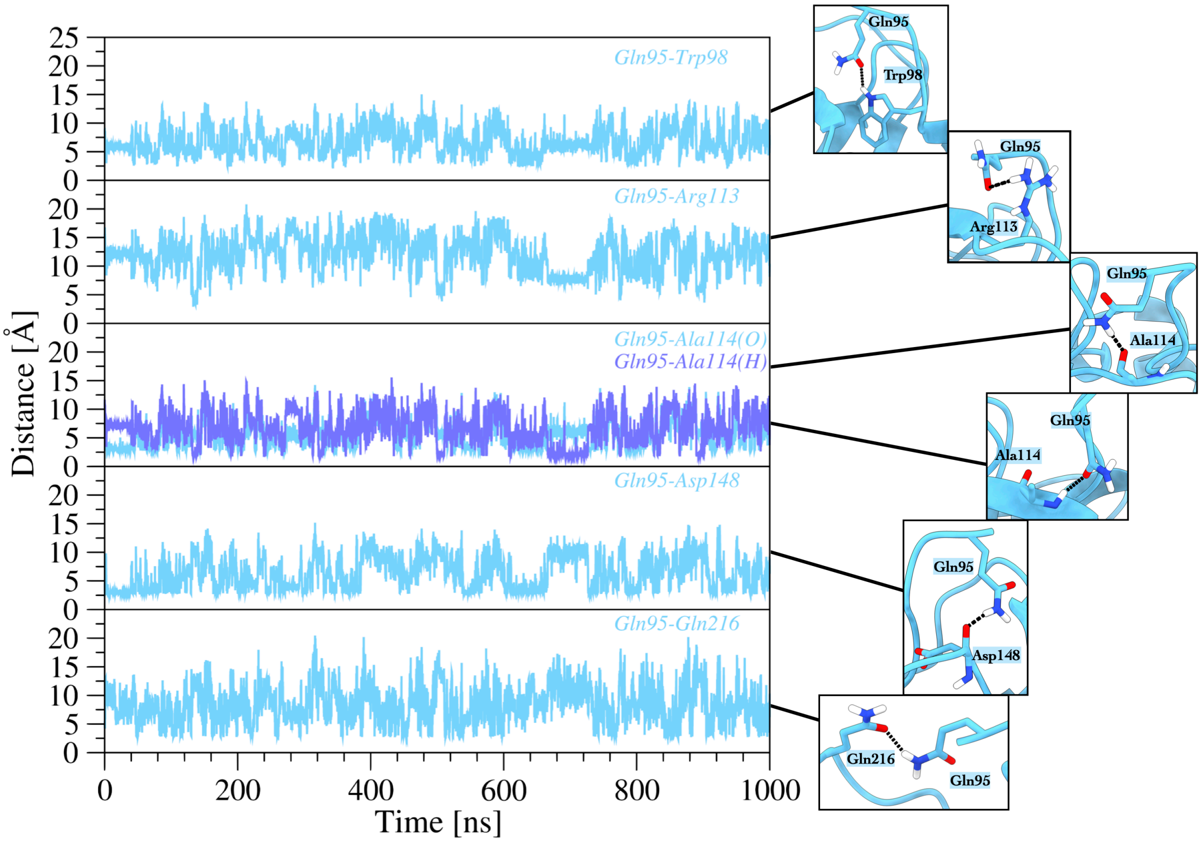

Fig. S2: Plot reporting the distances of the H-bonds established by Gln95 in the Leu95Gln TPP1 system. On the right side, insets displaying representative frames in which the aforementioned H-bonds are formed.

Supplementary Note 3: Complementary data on the time-series analysis

In the following section, we display our complementary PCA analyses on the WT TPP1, Glu169Δ TPP1, Lys170Δ TPP1, and Leu95Gln TPP1 MD simulations. Herein, we report the porcupine plot associated with first eigenvector (i.e. carrying the highest amount of variance), the projection of the module of the second eigenvector, and the associated porcupine plot. Moreover, we show the scree plot of the eigenvalues as measured by carrying out PCA on the 3 replicas. For the sake of clarity, we also report the percentages of variance in the Tab. S2

SYSTEM	Replica 1 (r1)	Replica 2 (r2)	Replica 3 (r3)
WT TPP1	50% (PC2:14%)	57% (PC2: 10%)	34% (PC2: 14%)
Glu169Δ TPP1	55% (PC2: 11%)	28% (PC2: 17%)	30% (PC2: 14%)
Lys170Δ TPP1	29% (PC2: 15%)	37% (PC2: 14%)	42% (PC2: 17%)
Leu95Gln TPP1	30% (PC2: 18%)	41% (PC2: 12%)	68% (PC2: 5%)

Tab. S2: Table displaying the percentage of variance carried out by the first eigenvector PC1 in each replica of the systems WT TPP1, Glu169Δ TPP1, Lys170Δ TPP1, and Leu95Gln TPP1. For the sake of clarity, we also reported (in brackets) the percentage of variance carried by the second eigenvector PC2.

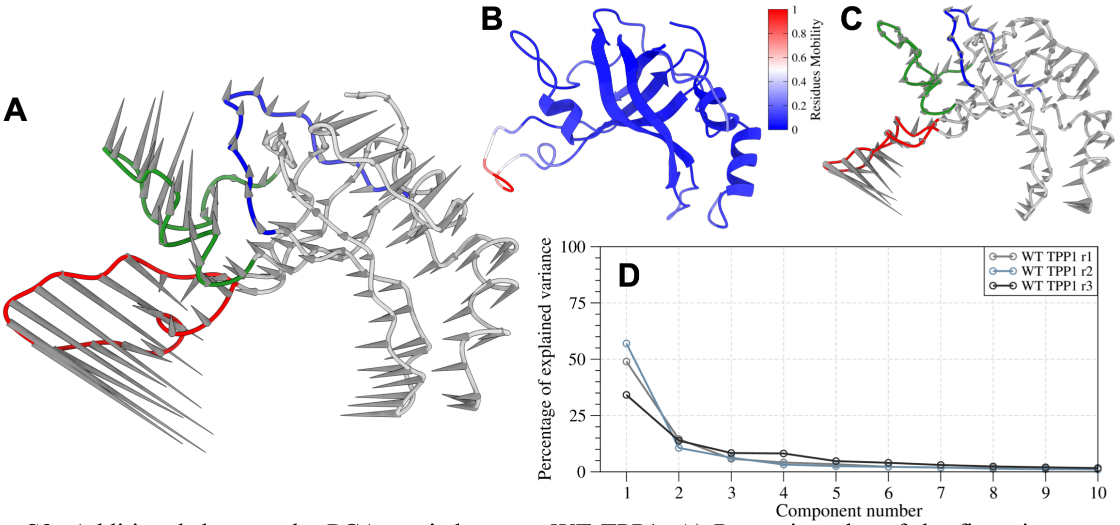

Fig. S3: Additional data on the PCA carried out on WT TPP1. **A**) Porcupine plot of the first eigenvector. **B**) Projection of module of the second eigenvector computed for each residue of WT TPP1. **C**) Porcupine plot of the second eigenvector. **D**) Scree plot displaying the percentage of explained variance carried out by the eigenvectors for each WT TPP1 replica.

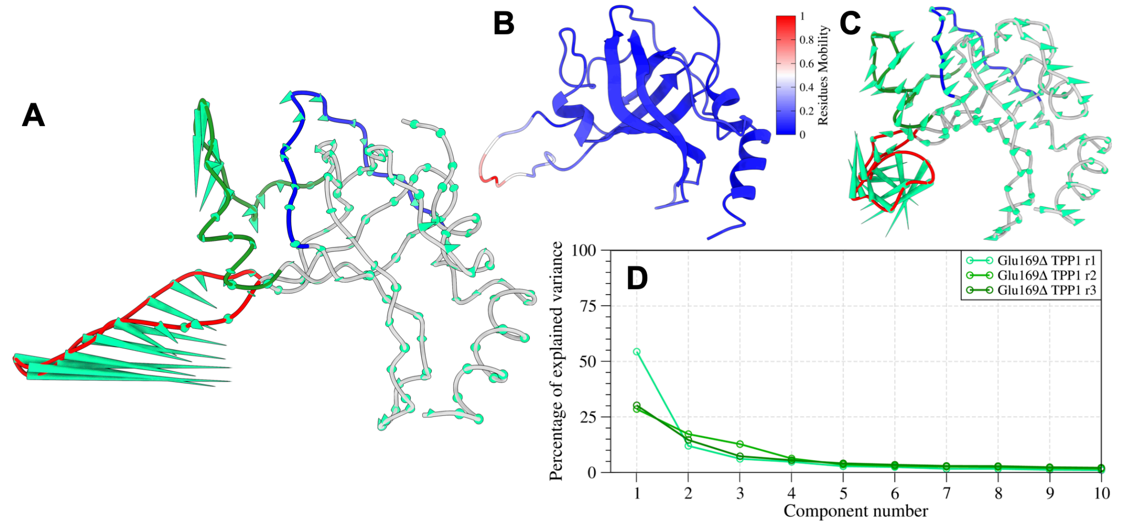

Fig. S4: Additional data on the PCA carried out on Glu169Δ TPP1. **A**) Porcupine plot of the first eigenvector. **B**) Projection of module of the second eigenvector computed for each residue of Glu169Δ TPP1. **C**) Porcupine plot of the second eigenvector. **D**) Scree plot displaying the percentage of explained variance carried out by the eigenvectors for each Glu169Δ TPP1 replica.

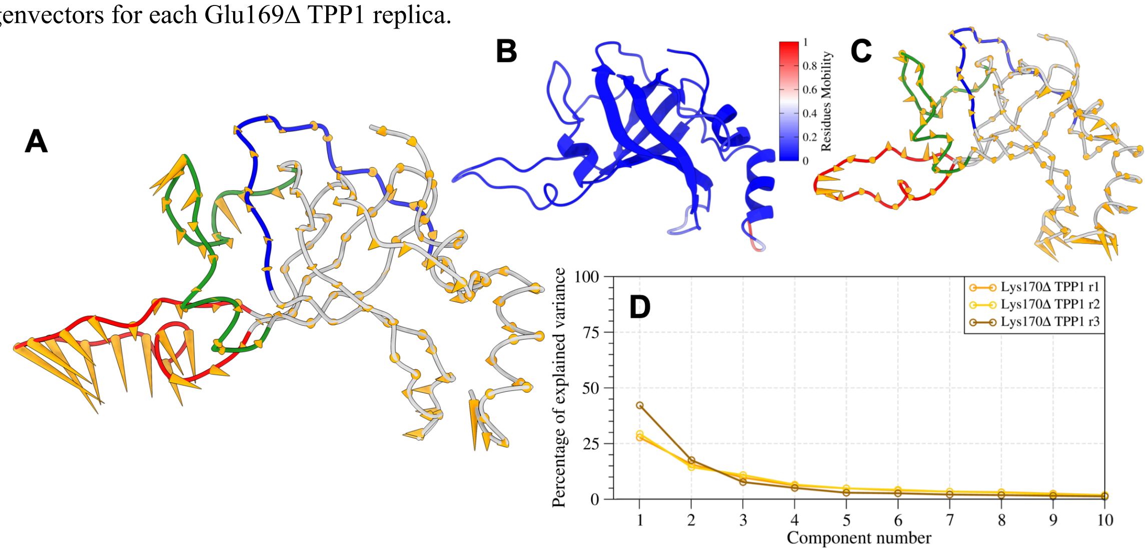

Fig. S5: Additional data on the PCA carried out on Lys170Δ TPP1. **A**) Porcupine plot of the first eigenvector. **B**) Projection of module of the second eigenvector computed for each residue of Lys170Δ TPP1. **C**) Porcupine plot of the second eigenvector. **D**) Scree plot displaying the percentage of explained variance carried out by the eigenvectors for each Lys170Δ TPP1 replica.

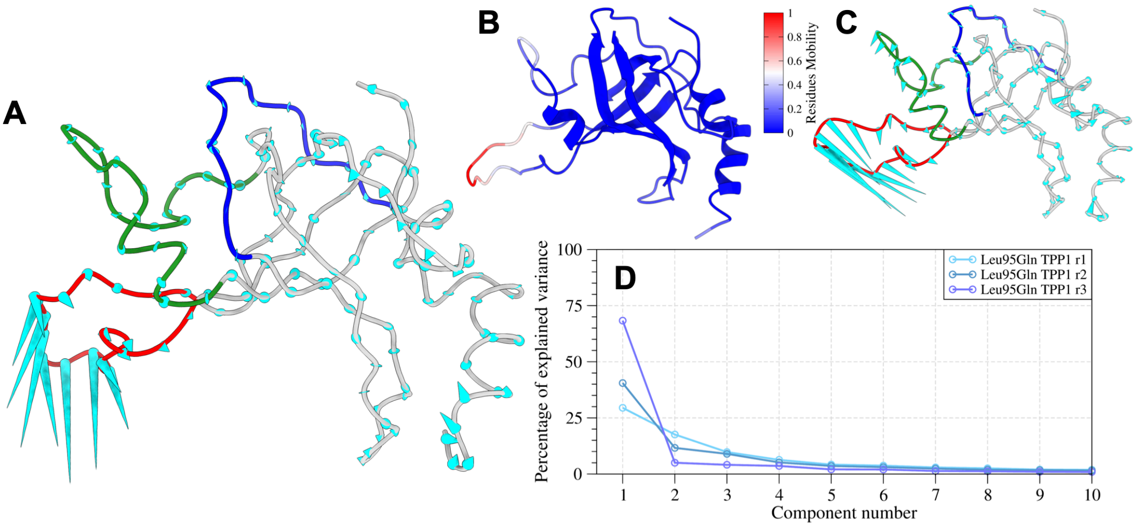

Fig. S6: Additional data on the PCA carried out on Leu95Gln TPP1. **A**) Porcupine plot of the first eigenvector. **B**) Projection of module of the second eigenvector computed for each residue of Leu95Gln TPP1. **C**) Porcupine plot of the second eigenvector. **D**) Scree plot displaying the percentage of explained variance carried out by the eigenvectors for each Leu95Gln TPP1 replica.

Supplementary Note 4: Protein structure network of the WT TPP1, Glu169Δ TPP1, Lys170Δ TPP1, and Leu95Gln TPP1 monomers

In this section, we reported our analysis of the WT TPP1's, Glu169Δ TPP1's, Lys170Δ TPP1's, and Leu95Gln's Protein Structure Network (PSN). In PSN, the residues are considered as nodes in a graph, interconnected by edges with attributed weights that are computed based on the non-covalent atomistic contacts established between nodes. In such a way, it is possible to compare graphs representing the same protein at different conditions. We have exploited this possibility by resolving the exclusive paths of TPP1 in the different systems, that is the identification of inter-residue interactions in TPP1 that are only present in the WT and in the Glu169Δ/Lys170Δ/Leu95Gln variants. As shown in Fig. S6 reported below, the PSN analysis agrees with the results obtained through the calculation of Pearson coefficients reported in Fig. 3, confirming the loss of most of the inter-residue interactions between the TEL patch and the Asp123-Gly141 overhang in the TPP1 mutants with respect to the WT.

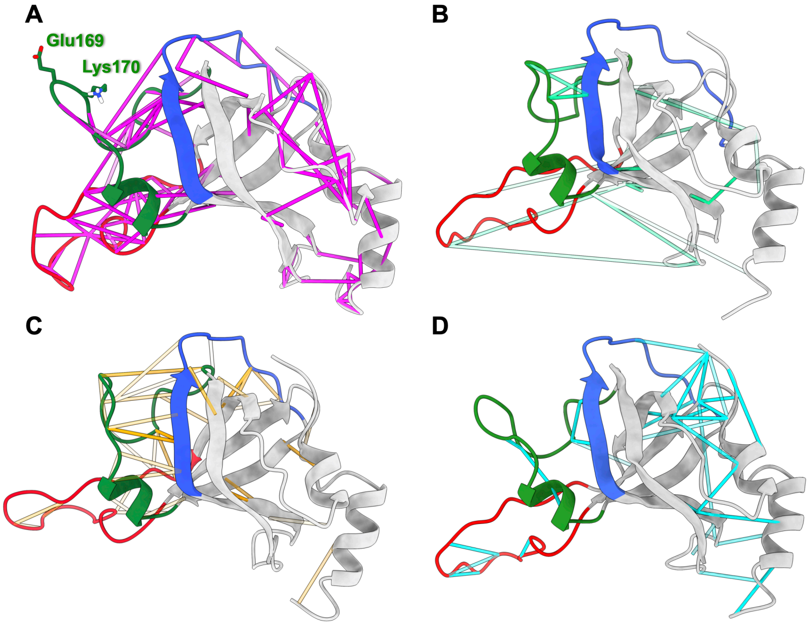

Fig. S7: Graph-based network analysis of the monomeric structure of WT TPP1, Glu169Δ TPP1, Lys170Δ TPP1, and Leu95Gln TPP1 during MD simulations. (**A**-**D**) The communications between the residues are projected upon the 3D protein structure of WT TPP1 (**A**), Glu169Δ TPP1 (**B**), Lys170Δ TPP1 (**C**), and Leu95Gln TPP1 (**D**). The three relevant moieties of the TPP1's OB domain ((**I**) Asp123-Gly141 overhand motif; (**II**) TEL-patch Knuckle motif; (**III**) TEL-patch Barrel part) are coloured following the color scheme reported in Fig. 3 of the original manuscript. The graph edges of WT TPP1 are coloured in magenta, the edges for Glu169Δ TPP1 are coloured in green, the edges for Lys170Δ TPP1 are in orange, while the edges of Leu95Gln TPP1 are coloured in cyan. Transparency is proportional to the weight of the edge, i.e. solid edges correspond to highly frequent connections.

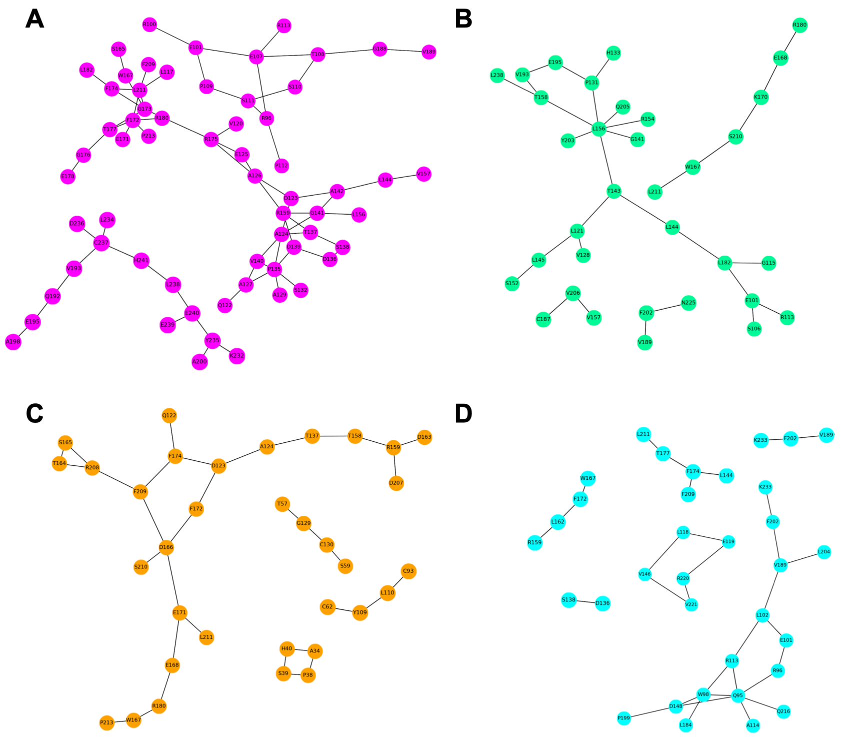

Fig. S8: Graph-based network analysis of the monomeric structure of WT TPP1, Glu169Δ TPP1, Lys170Δ TPP1, and Leu95Gln TPP1 during MD simulations. (**A-D**) Representation of the graphs corresponding to WT TPP1, Glu169Δ TPP1, Lys170Δ TPP1, and Leu95Gln TPP1 respectively. The graphs herein reported are generated to best represent the connection between nodes and are unrelated to the protein 3D structure.

Supplementary Note 5: List of TPP1 mutations

Table S3 reports the known TPP1 mutations at their binding interface with TERT with the corresponding phenotypes and references.

TPP1's mutations	Phenotype	Reference
Leu95Gln	Severe telomeres shortening	[1]
Glu169Δ	Reduced telomeres length-HHS	[2-3]
Lys170Δ	Reduced telomeres length-HHS	[4-6]

Tab. S3: Tables reporting the TPP1 mutations involving residues the interface of the WT TPP1-hTEN dimer interface (as depicted in PDB ID: 7TRE). The impact of all these mutations on the heterodimer stability was verified through docking calculations and extensive MD simulations. For each mutation, we indicated both the corresponding experimental phenotype and the related references.

Supplementary Note 6: Additional data on the heterodimers MD simulations

In this section, we show complementary information about the MD simulations carried out on the TPP1-TERT heterodimers (i.e. WT TPP1-TERT, Glu169Δ TPP1-TERT, Lys170Δ TPP1-TERT, and Leu95Gln TPP1-TERT). Notably, Leu95Gln TPP1 is characterised by the establishment of intra-protein H-bonds along the simulation that led to the formation of a short α-helix located at the N-terminus of the OB-domain. Such secondary structure element is driven by the charge-enforced H-bond between the side-chains of Gln95 and TERT's Glu648, and further stabilised by the H-bonds between the backbones of Gln95, Gly91, and Arg92 (Fig. S8C-D). A further element of stability is endowed by the charge-enforced H-bond between the side-chain of Arg113 and the carbonyl oxygen of Arg96. All these interactions, unique of this mutant, affect the orientation of Leu95Gln TPP1 with respect to TERT's hTEN, and drive it away from the physiological 7TRE's conformation.

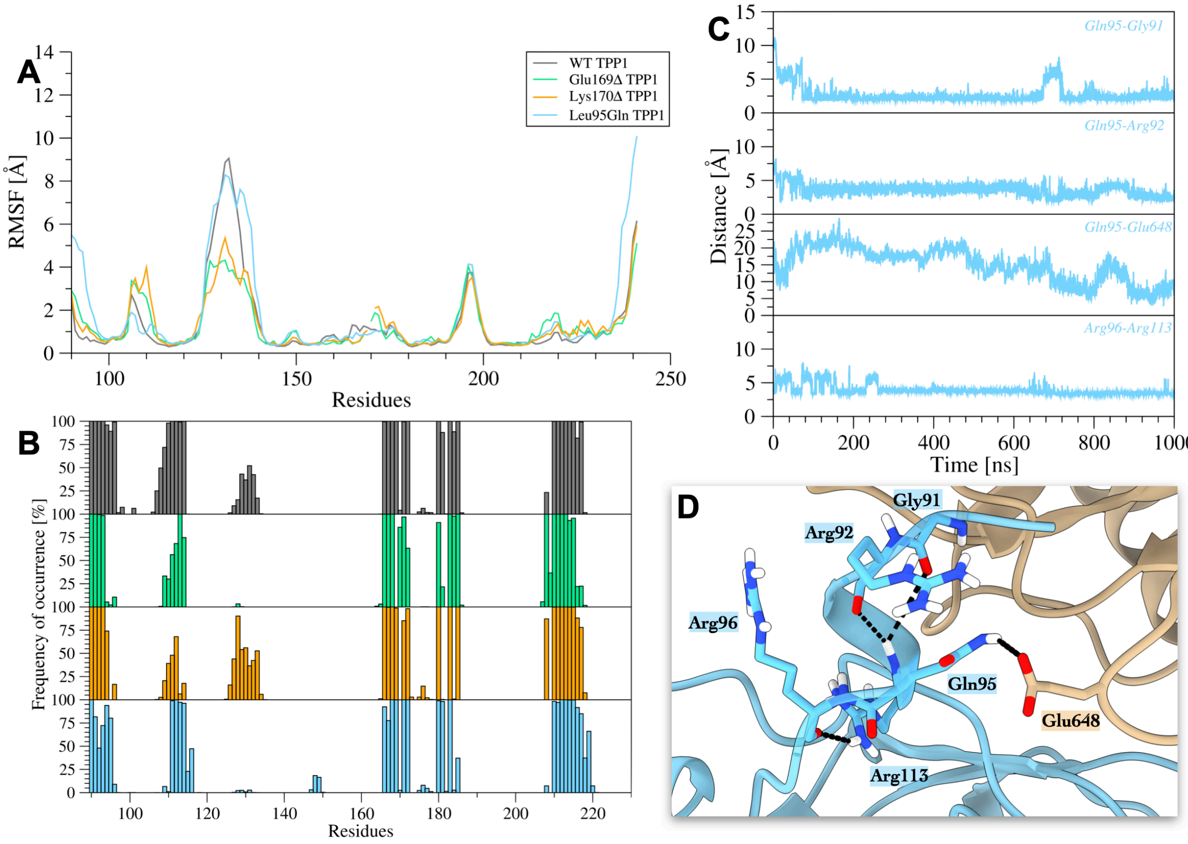

Fig. S9: Additional information on the MD simulations carried out on the WT TPP1-TERT, Glu169Δ TPP1-TERT, Lys170Δ TPP1-TERT, and Leu95Gln TPP1-TERT heterodimers. **A**) Plot of RMSF values computed for each residue of WT, Glu169Δ, Lys170Δ TPP1, and Leu95Gln TPP1 (grey, green, orange, and cyan respectively). **B**) Histogram reporting the frequency of occurrence of the contacts between TERT and the several TPP1 variants (see "Methods" for details). **C**) Plot reporting the distances of the H-bonds established by Gln95 in the Leu95Gln TPP1-TERT system. **D**) Panel displaying a representative frame of the Leu95Gln TPP1-TERT MD simulation in which the aforementioned H-bonds are formed.

Supplementary Note 7: Contacts ruling the TPP1-TERT protein-protein interaction

Herein, we reported plots displaying the time evolutions of the pivotal contacts ruling the protein-protein interactions between TPP1 and TERT. We collected such data for the WT TPP1-TERT, Glu169Δ TPP1-TERT, Lys170Δ TPP1-TERT, and Leu95Gln TPP1-TERT systems.

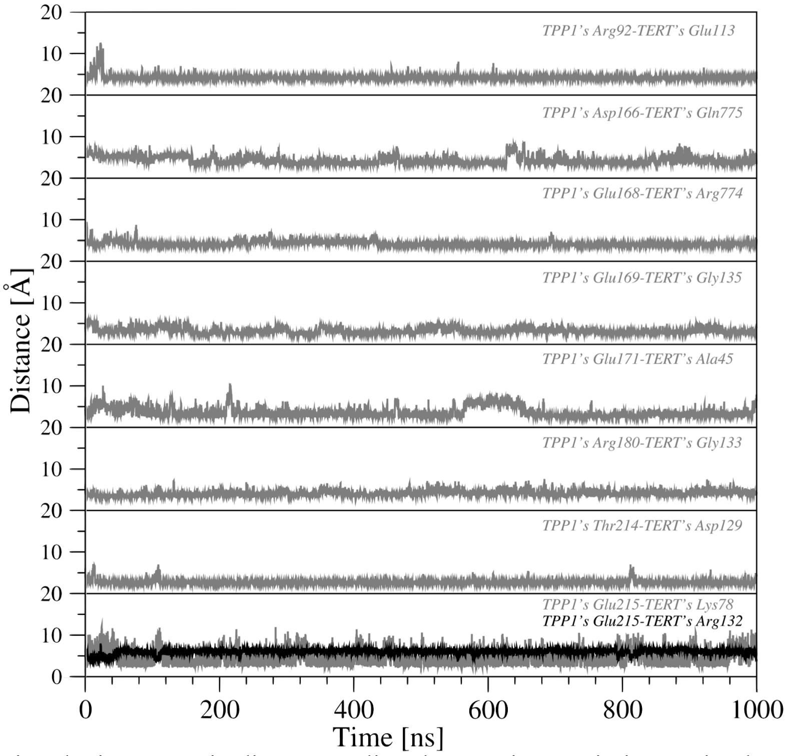

Fig. S10: Plot reporting the inter-protein distances ruling the protein-protein interaction between WT TPP1 and TERT in the heterodimer simulation.

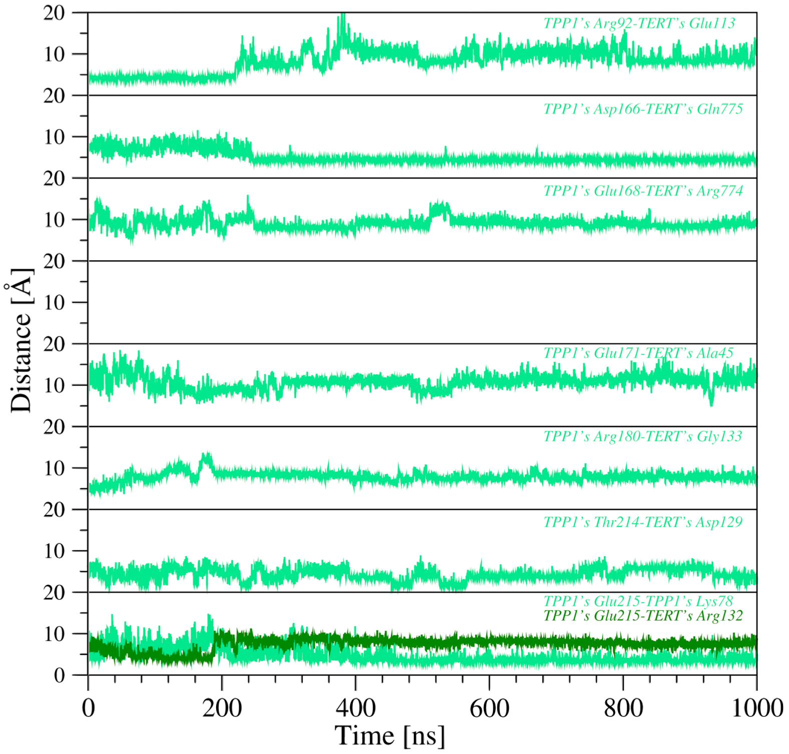

Fig. S11: Plot reporting the inter-protein distances ruling the protein-protein interaction between Glu169Δ TPP1 and TERT in the heterodimer simulation.

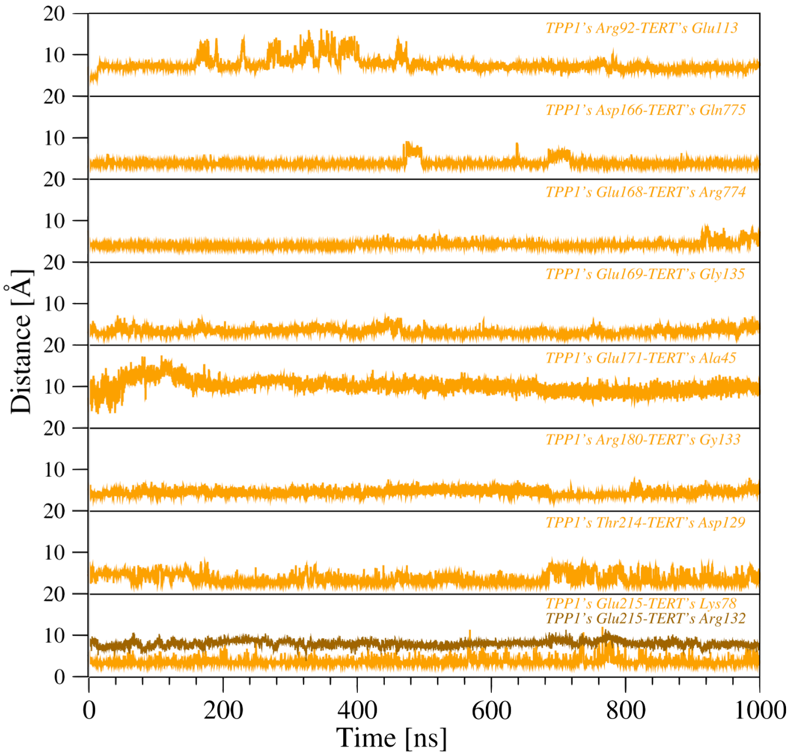

Fig. S12: Plot reporting the inter-protein distances ruling the protein-protein interaction between Lys170Δ TPP1 and TERT in the heterodimer simulation.

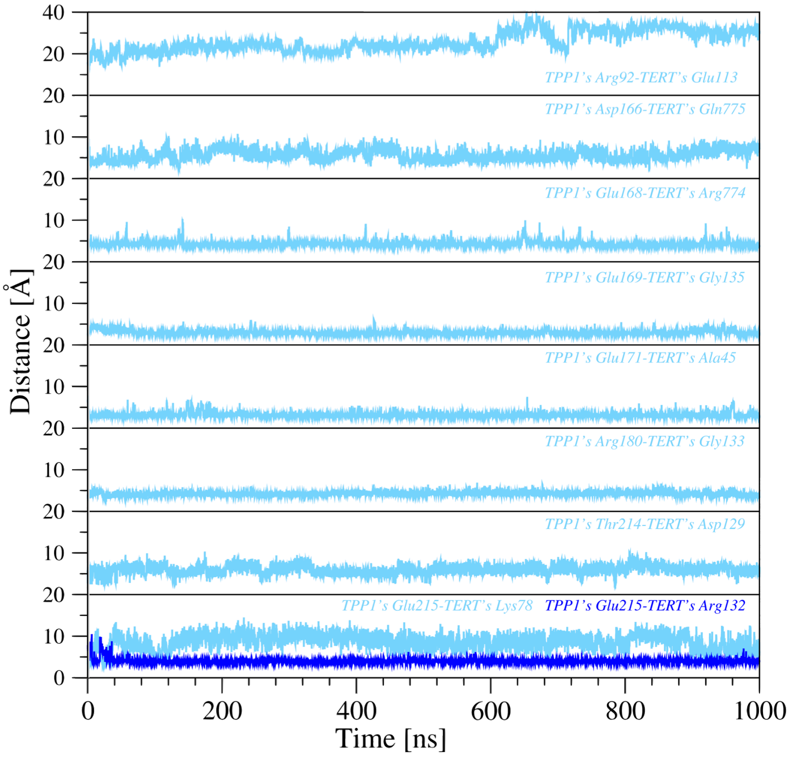

Fig. S13: Plot reporting the inter-protein distances ruling the protein-protein interaction between Leu95Gln TPP1 and TERT in the heterodimer simulation.

Supplementary Note 8: Details on systems building

In this section, we show the total number of atoms (including water molecules and salt ions) of the systems investigated in the present work.

System	Number of atoms
WT TPP1 *(monomer in solution)*	41320
Glu169Δ TPP1 *(monomer in solution)*	50999
Lys170Δ TPP1 *(monomer in solution)*	48827
Leu95Gln TPP1 *(monomer in solution)*	50980
WT TPP1-TERT *Complex*	284904
Glu169Δ TPP1-TERT *Complex*	280705
Lys170Δ TPP1-TERT *Complex*	286498
Leu95Gln TPP1-TERT *Complex*	298887

Tab. S4: Tables reporting the amount of atoms for each system we investigated. The numbers displayed in the column on the right take also in account both water molecules (TIP3) and salt ions (NaCl).

SUPPLEMENTARY REFERENCES

1. Tummala H, Collopy LC, Walne AJ, Ellison A, Cardoso S, Aksu T et al. (2018) Homozygous OB-fold variants in telomere protein TPP1 are associated with dyskeratosis congenita–like phenotypes. Blood 132:1349-1353.
2. Henslee G, Williams CL, Liu P, Bertuch AA (2021) Identification and characterization of novel ACD variants: modulation of TPP1 protein level offsets the impact of germline loss-of-function variants on telomere length. Cold Spring Harb Mol Case Stud 7:a005454.
3. Revy P, Kannengiesser C, Bertuch AA (2022) Genetics of human telomere biology disorders. Nature Reviews Genetics 24:86-108.
4. Kocak H, Kocak H, Ballew BJ, Bisht K, Eggebeen R , Hicks BE et al. (2014) Hoyeraal-Hreidarsson syndrome caused by a germline mutation in the TEL patch of the telomere protein TPP1. Genes Dev. 28:2090-2102.
5. Guo Y, Kartawinata M, Li J, Pickett HA, Teo J, Kilo T et al. (2014) Inherited bone marrow failure associated with germline mutation of *ACD*, the gene encoding telomere protein TPP1. Blood 124:2767-2774.
6. Bisht K, Smith EM, Tesmer VM, Nandakumar J (2016) Structural and functional consequences of a disease mutation in the telomere protein TPP1. Proc. Natl Acad. Sci. USA 113:13021-13026.
