# Supplementary material for: Conformational plasticity and allosteric communication networks explain Shelterin protein TPP1 binding to human telomerase
Source: Commun Chem. 2023 Nov 7;6:242. doi: 10.1038/s42004-023-01040-y (PMC10630336; doi:10.1038/s42004-023-01040-y)
Supplement: Supplementary file 3 — Description of Additional Supplementary Files [file 42004_2023_1040_MOESM3_ESM.pdf]

Description of Additional Supplementary Files

File name: Supplementary Data 1

Description: PDB of the TPP1 structures W1, W2, W3, ΔE1, ΔE2, ΔK1, ΔK2, LQ1, and LQ2

File name: Supplementary Video 1

Description: video detailing 485 the effect of Lys170Δ on TPP1 functional dynamics
